# A Challenge-Response Assisted Authorisation Scheme for Data Access in Permissioned Blockchains

**DOI:** 10.3390/s20174681

**Published:** 2020-08-19

**Authors:** Xiaoshuai Zhang, Chao Liu, Kok Keong Chai, Stefan Poslad

**Affiliations:** School of Electronic Engineering and Computer Science, Queen Mary University of London, London E1 4NS, UK; c.liu@qmul.ac.uk (C.L.); michael.chai@qmul.ac.uk (K.K.C.); stefan.poslad@qmul.ac.uk (S.P.)

**Keywords:** privacy enhancement, permissioned blockchain, access control, decentralised network

## Abstract

Permissioned blockchains can be applied for sharing data among permitted users to authorise the data access requests in a permissioned blockchain. A consensus network constructed using pre-selected nodes should verify a data requester’s credentials to determine if he or she have the correct permissions to access the queried data. However, current studies do not consider how to protect users’ privacy for data authorisation if the pre-selected nodes become untrusted, e.g., the pre-selected nodes are manipulated by attackers. When a user’s credentials are exposed to pre-selected nodes in the consensus network during authorisation, the untrusted (or even malicious) pre-selected nodes may collect a user’s credentials and other private information without the user’s right to know. Therefore, the private data exposed to the consensus network should be tightly restricted. In this paper, we propose a challenge-response based authorisation scheme for permissioned blockchain networks named Challenge-Response Assisted Access Authorisation (CRA^3^) to protect users’ credentials during authorisation. In CRA^3^, the pre-selected nodes in the consensus network do not require users’ credentials to authorise data access requests to prevent privacy leakage when these nodes are compromised or manipulated by attackers. Furthermore, the computational burden on the consensus network for authorisation is reduced because the major computing work of the authorisation is executed by the data requester and provider in CRA^3^.

## 1. Introduction

Permissioned blockchain networks stem from blockchain [1,2] as a decentralised network structure used for trading or sharing data among permitted users (nodes). Permissioned blockchain networks have been applied in numerous fields, including security services [3,4], Internet of Things (IoT) [5,6], and reputation systems [7,8]. In a permissioned blockchain network, there are numerous pre-selected nodes that constitute the consensus network to realise authorisation that tolerate Byzantine faults, avoiding a single point of failure and providing system scalability [9,10].

In current studies, pre-selected consensus nodes are normally assumed to be trusted in order to authorise the data access and to transport users’ private data in a consensus network [11]. However, one important issue that has not been considered is that users’ private data can be utilised by the pre-selected nodes in the consensus network during authorisation, since these nodes may be manipulated by the attacker to be untrusted (malicious). To be specific, in many current designs of authorisation for permissioned blockchains [10,12], the nodes in a consensus network require the use of many users’ private information (e.g., credentials and personal information) to authorise their data access requests. When some nodes are compromised by an attacker, the users’ private information can be disclosed to an attacker without any barriers.

Furthermore, if pre-selected nodes in the consensus network are malicious, such nodes can exploit a user’s private data to analyse his or her behaviour without such a user’s right to know that this violates their privacy. For example, when a permissioned blockchain network is applied to smart power grids or smart charging, the behaviour of the registered user can be analysed based upon the uploading time and length of his or her electric bills if the nodes are malicious or compromised in the consensus network [13,14]. Similarly, in an eHealth scenario, a patient’s private information could be leaked with respect to the above situation.

However, research regarding privacy protection for authorisation in a permissioned blockchain is currently in its infancy. Many studies does not consider how to protect users’ private information during authorising data access in permissioned blockchains, as they regard the consensus network (pre-selected nodes) as a fully trusted part. Even though some recent studies (e.g., [15]) start to raise concerns protecting users’ private information in the authorisation of a permissioned blockchain, such methods may leak users’ private information to particular nodes in the consensus network of the permissioned blockchain. Therefore, in a permissioned blockchain, the protection of users’ private information is still an open problem to be addressed for the authorisation of data access by the untrusted consensus network.

In this paper, our major novelty lies in considering privacy protection for the requisite authorisation so as to avoid privacy leakage when some nodes of the consensus network are untrusted (malicious or compromised) in a permissioned blockchain. A Challenge-Response Assisted Access Authorisation (CRA^3^) scheme based on the challenge-response mechanism is proposed to protect users’ private information during the authorisation for data access. The contributions of this paper are summarised as follows.

- Unlike many mainstream designs [10,12,16] that cannot protect users’ private information in the authorisation, CRA^3^ can realise authorisation without revealing the access permissions and other private information of users to nodes in the consensus network and keep users anonymous in the permissioned blockchain because we consider the consensus network as untrusted.
- A theoretical security model and proof are illustrated formally to show that CRA^3^ can achieve the confidentiality of indistinguishability under chosen-ciphertext attacks (IND-CCA) [17] to avoid privacy leakage during the authorised data access in a permissioned blockchain.
- Compared with [12,16], the communication overhead decreases because the size of the information needed for the authorisation is much smaller in our designed algorithms.
- Most of the computing work for authorising the data access request is executed by the data requester and provider to decrease the transaction cost and the burden on the consensus network.

The rest of this paper is organised as follows. The related work is presented in Section 2 and the preliminaries are introduced in Section 3 to help understand our concrete algorithms for our CRA^3^ scheme. Then, the problem we try to solve and the algorithm definitions of our CRA^3^ scheme are described in Section 4. After that, our proposed CRA^3^ scheme is demonstrated in Section 5, followed by a theoretical security analysis in Section 6. Furthermore, the experiments and the comparison of the results are demonstrated in Section 7 to analyse the performance of CRA^3^ in terms of the computational time consumption, the communication overhead, and the transaction fee for authorisation. Finally, we conclude our work in Section 8.

## 2. Related Work

The authors in [12] presented a personal data transmission scheme via blockchain consensus networks. In their scheme, the stored personal data are transmitted by a blockchain with constructed access policies to authorise data access via a consensus network. As they regard the consensus network as a trusted network, all of the users’ private data (including access permissions) are exposed to the consensus network in their scheme. If some nodes in the consensus network are compromised, the transmitted personal data can be revealed to attackers. The Healthcare Data Gateways scheme (HDG) [16] has a similar issue in the gateway (consensus part) design; users’ access tokens can be accessed via the gateway without any secure precaution.

Reference [18] discusses several applications of blockchain in IoT scenarios have been discussed including wireless software updates, smart charging, and car sharing services. However, there is no consideration for privacy protection in the consensus network when blockchains are applied to the above scenarios. A transaction framework for permissioned blockchains with a group policy was proposed, but the group policy was determined only by computing power without any data access control for users [19].

In the smart grid area, the authors in [20] proposed how to build permissioned blockchain networks for bill collection and power load adjustment. There are two reasons for utilising permissioned blockchain networks: first, the decentralisation feature of blockchain can achieve a fail-safe state to avoid a single point of failure; second, the structure of permissioned networks can block unauthenticated and unauthorised access. However, when some nodes are manipulated by the attackers in a consensus network, the solution to avoid privacy leakage is not discussed. More recently, an energy trading system supported by a permissioned blockchain was proposed by Gai et al. [15], where message validation was considered for a scenario of smart grids. The authors utilised group signature [21] to construct an identity validation algorithm to validate the edge nodes (and their messages) during the activities defined by the smart contracts. However, for the process of identity validation, the leader of the permissioned nodes knows the real identity of each node in the system setup phase. Meanwhile, this trading system can only ensure data integrity (signature) of messages but cannot provide confidentiality protection because messages between two edge nodes are plain to all permissioned nodes without any encryption. Furthermore, the issue of key revocation [22,23] is quite complicated and is not considered in such a large-scale decentralised system [24].

## 3. Preliminaries

### 3.1. Permissioned Blockchain Networks

**Figure 1:**
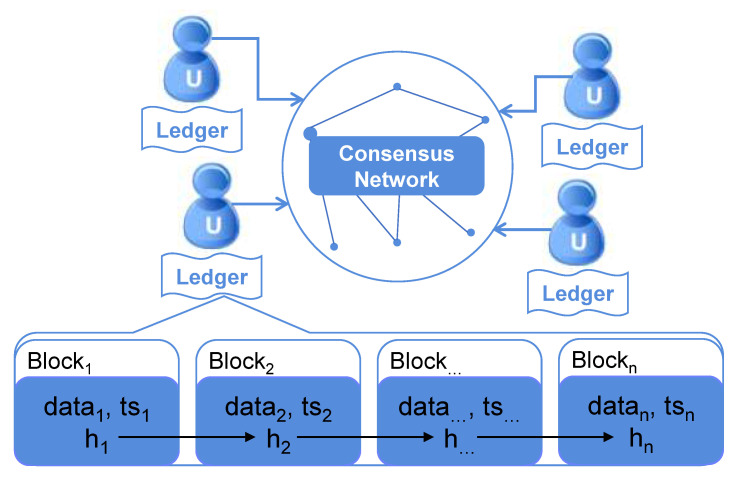
The structure of the permissioned blockchain network.

The network structure of a permissioned blockchain shown in Figure 1 is the same as that of a public blockchain. All the nodes are anonymous in the network. However, the data (ledger) in each node are private, which means the data access between the two nodes should be authorised to ensure one node has the permissions to access the data in another node.

Meanwhile, the private ledger of each node is a blockchain structure. Each block contains data, a cryptographic hash value (*h*) and a timestamp (*ts*) in the blockchain [1]. The hash value for establishing the link between two blocks is generated by the following rules:

$$h_{i}=\left\{\begin{array}{cc} Hash(data_{1}||ts_{1}) & ,\;i=1\\ Hash(h_{i-1}||data_{i}||ts_{i}) & ,\;i=2,\ldots ,n \end{array}\right.$$

### 3.2. Lagrange Interpolating Polynomial

Let *q* be a prime and

$$f\left(x\right)=a_{0}+a_{1}x+\ldots +a_{t-1}x^{t-1}\;\left(mod\;q\right)$$

with a polynomial of degree *t*, where $a_{0},a_{1},\ldots a_{t}\in Z_{q}$ are coefficients. Given any *t* points $\left\{\left(x_{1},y_{1}\right),\ldots ,\left(x_{t},y_{t}\right)\right\}$ on f(x), the coefficient $a_{0}=f\left(0\right)$ can be computed with Lagrange interpolating polynomial

$$f\left(0\right)=\sum_{i\in A}\Delta_{i}y_{i}\;\left(mod\;q\right),$$

where $\Delta_{i}=\prod_{j\in A/\{i\}}\frac{x_{j}}{x_{j}-x_{i}}\;\left(mod\;q\right)$ are the Lagrange interpolation coefficients and A={1,2,…,t}. Note that f(x) can be reconstructed with any *t* points based on polynomial theory; however, f(x) cannot be reconstructed (f(0) cannot be computed either) with any points fewer than *t* [25].

## 4. Problem and Definitions

In this section, we first describe our scheme model and the targeted security problem we try to address. Then, seven algorithms are defined to construct our proposed CRA^3^ scheme in the second part, which is followed by the security definitions including the correctness and confidentiality of our CRA^3^ as the last part.

### 4.1. Problem Statement

**Figure 2:**
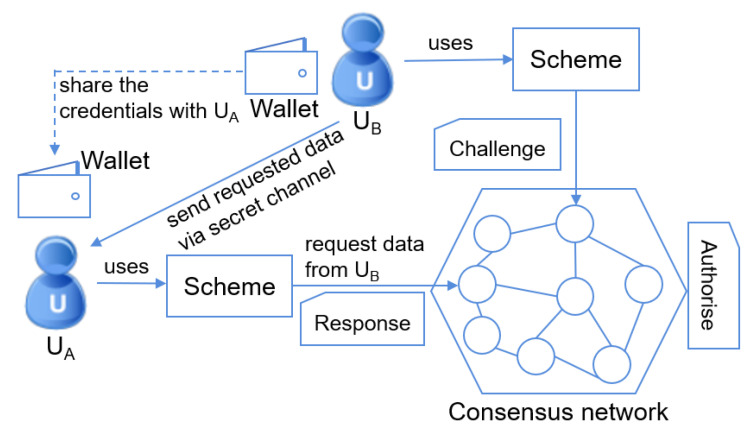
The system model of our proposed Challenge-Response Assisted Access Authorisation (CRA^3^) scheme.

The proposed scheme model is demonstrated in Figure 2 with three entities: two users (nodes) and the consensus network (constructed by pre-selected nodes in the permissioned blockchain network), denoted by $U_{A}$ (data requester), $U_{B}$ (data provider) and $CN_{pm}$, respectively. Since all the nodes in the network are anonymous (unknown identities), one node cannot trust another node in the permissioned blockchain network. Therefore, the consensus network $CN_{pm}$ is needed as a mediator to finish the validations in the challenge-response phase. If $U_{A}$ has the correct permissions to access his/her requested data, $U_{B}$ establishes a secret channel with $U_{A}$ to transport the encrypted data after the authorisation.

Since the nodes in $CN_{pm}$ are assumed to be untrusted, one untrusted node can output the incorrect result of the authorisation and collect the user’s private data from the communication in the challenge-response phase. If a node outputs the incorrect authorisation result, the consensus network can punish this dishonest node according to the applied consensus mechanism (e.g., Byzantine fault tolerance). On the other hand, if a malicious node collects the user’s data from the challenge-response communication and then attempts to reveal the user’s credentials (or other private information), our proposed scheme should prevent the private data leakage. Hence, the purpose of our proposed scheme is to authorise data access without involving the users’ credentials ($U_{id}=\left\{U_{1},U_{2},\ldots ,U_{n}\right\}$) whilst ensuring the transported data is confidential, i.e., $\forall M\in {\left\{0,1\right\}}^{*},C=f\left(M\right)$, and any probabilistic polynomial-time algorithm A computes *M* or $U_{id}$ with its advantage $Adv_{A}^{CN_{pri}}=Pr\left[c=M\vee c\subseteq U_{id}|c=A\left(C,D_{Ch},D_{Re}\right)\right]<\varepsilon$, where *M* is plaintext data, *C* is encrypted data that transmitted between $U_{A}$ and $U_{B}$, $D_{Ch}$ and $D_{Re}$ denote the data used in the processes *Challenge* and *Response*, respectively, and ε represents a negligible probability.

### 4.2. Scheme Definitions

In this section, we introduce the credentials used in the authorisation and define all of the seven algorithms that comprise our proposed CRA^3^ scheme.

Credentials for authorisation: The credentials used for authorisation are the identity attributes. For instance, in the hospital scenario, if a doctor wants to request a patient’s medical records, the identity attributes can be the patient’s name, age, medical record number, social number, and so on. We assume that in reality, the patient has shared the identity attributes and a unique reference number to identify the needed identity attributes for the doctor’s data request before inquiring about the data. We denote unique reference number (key) and the identity attributes (values) by Rn and the sequence $AT^{v}=\left\{AT_{1}^{v},AT_{2}^{v},\ldots ,AT_{n}^{v}\right\}$, respectively.

- Setup (λ): This algorithm takes the security parameter λ and generates the public parameter pp.
- Request (pp): $U_{A}$ uses the algorithm to send a data access request *Q* to $U_{B}$ via $CN_{pm}$.
- Challenge (pp,Q): $U_{B}$ constructs and sends the challenge Ch to $U_{A}$ with the identity attributes sequence $AT^{v}$.
- Response (pp,Ch): $U_{A}$ uses its own sequence $AT^{\prime v}$ to calculate and send the response Re to the $CN_{pm}$.
- Authorise (Ch, Re): $CN_{pm}$ validates the correctness of the response Re based upon the challenge Ch.
- Encrypt ($pp,M,AT^{v}$): $U_{B}$ uses this algorithm to encrypt the requested data *M* then return the ciphertext *C* and a point *P* (for decryption use) to $U_{A}$.
- Decrypt (pp,C, *P*, $AT^{\prime v}$): $U_{A}$ decrypts the encrypted data *C* to retrieve the requested data *M* with the given point *P*.

### 4.3. Security Definition

The definitions of correctness and the IND-CCA (indistinguishability under chosen-ciphertext attacks) security (confidentiality) for our CRA^3^ scheme are illustrated as follows.

#### 4.3.1. Correctness

For any pp←*Setup(λ)* and any plaintext $M\in {\left\{0,1\right\}}^{*}$, the CRA^3^ scheme is correct if $Decrypt(pp,C,AT^{\prime v})=M$ always holds, where $C=Encrypt(pp,M,AT^{v})$.

#### 4.3.2. Confidentiality (IND-CCA Security)

Formally, the adversary defined to prove the theoretical security of our proposed CRA^3^ scheme is: *Type-IND adversary*.

*Type-IND adversary*: In the *Authorise* phase, the adversary cannot determine the message that the given challenge ciphertext is encrypted from, even though the sequence of the identity attributes $AT^{v}$ is revealed to the adversary.

**Game 1.** *Let $A_{1}$ be the given Type-IND adversary, and the index of the target data provider be t (1⩽t⩽n). The game played by the challenger C and the adversary $A_{1}$ is described with the following five phases:*

- *Initialise:*
  C first generates the public parameter pp via running the algorithm Setup. Then, C generates *n* data providers (key-value pairs) $\left\{Rn^{i},AT^{v_{i}}\right\}\;\left(1⩽i⩽n\right)$ and the target data provider is $\left\{Rn^{t},AT^{v_{t}}\right\}$. The generated pp and all $Rn^{i}\;\left(1⩽i⩽n\right)$ are given to the adversary $A_{1}$.
- *Queries:*
  The following queries can be requested by $A_{1}$ for polynomial times in the game:
  1. *Identity attributes query (i)*: C responds with the sequence of the random identity attributes $rAT^{v_{i}}$;
  2. *Encrypt query(M,i)*: C outputs the ciphertext $C=Encrypt(pp,M,AT^{v_{i}})$ and the point *P* on the constructed polynomial f(x) in the *Encrypt* phase;
  3. *Decrypt query(C,P,i)*: C decrypts *C* via running the algorithm *Decrypt*, then responds with the plain message.
- *Challenge:*
  $A_{1}$ submits two equal-length messages $M_{0}^{*}$ and $M_{1}^{*}$. C picks $\rho \in_{R}\left\{0,1\right\}$, and then computes and returns the challenge ciphertext $C^{*}=Encrypt\left(pp,M_{\rho }^{*},AT^{v_{i}}\right)$.
- *Constraints:*
  1. $\left(M_{0}^{*},t\right)$ and $\left(M_{1}^{*},t\right)$ are not allowed to appear in the above *Encrypt query*;
  2. The target data provider’s index *t* and the challenge ciphertext $C^{*}$ are not allowed to appear in the above *Decrypt query*.
- *Guess:*
  $A_{1}$ can win the game if its output $\rho^{\prime }\in_{R}\left\{0,1\right\}$ satisfies the condition $\rho =\rho^{\prime }$.
  Now, the advantage of $A_{1}$ could be defined as:
  $$Adv_{A_{1}}^{IND-CCA}\left(\lambda \right)=|Pr\left[\rho =\rho^{\prime }\right]-\frac{1}{2}|.$$

Note that the probability analysis is presented after the *Guess* phase in the formal confidentiality proof of our CRA^3^ scheme.

**Definition** **1** (IND-CCA Security). *The CRA^3^ scheme is IND-CCA secure if the advantage $Adv_{A_{1}}^{IND-CCA}\left(\lambda \right)$ of any probabilistic polynomial-time adversary $A_{1}$ is negligible.*

## 5. Proposed Scheme CRA^3^

In this section, we illustrate our proposed CRA^3^ scheme (Challenge-Response Assisted Access Authorisation) with the seven algorithms defined in Section 4.2, including Setup, Request, Challenge, Response, Authorise, Encrypt, and Decrypt. In CRA^3^, *AES* (Advanced Encryption Standard [26]) is used to encrypt the requested data and the Lagrange interpolating polynomial is utilised to construct challenge-response authorisation and protect the encrypting/decrypting key of *AES*.

- **Setup (λ):**
  This procedure outputs public parameters pp with the security parameter λ using the following steps.
  1. Generate a big prime *q* ($q>2^{\lambda }$);
  2. Select one secure cryptographic hash function $H:{\left\{0,1\right\}}^{*}\to {\left\{0,1\right\}}^{\lambda }$;
  3. Select a symmetric encryption algorithm, e.g., AES (Advanced Encryption Standard);
  4. Output the public parameters pp=(q,H,AES).
- **Request (pp):**
  The user $U_{A}$ (data inquirer) prepares the query *Q* via the following steps.
  1. Decide on the data to be requested. Note that $U_{A}$ should have the corresponding identity attributes (a sequence, $AT^{\prime v}$) and the unique reference number ($R_{n})$ that is shared by $U_{B}$. For illustrating the remaining parts of the proposed scheme, we assume the requested data is in one block $B_{id}$;
  2. Prepare the unique reference number Rn then send the request $Q=(B_{id},Rn)$ to $U_{B}$ through $CN_{pm}$.
- **Challenge (pp,Q):**$U_{B}$ generates the challenge Ch based upon the request *Q* from $U_{A}$ via the following steps.
  1. Prepare the sequence of the identity attributes (values): $AT^{v}=\left\{AT_{1}^{v},AT_{2}^{v},\ldots ,AT_{n}^{v}\right\}$ based upon the unique reference number Rn∈Q;
  2. Calculate the hash value of each element in the sequence $AT^{v}$ to get the sequence $AH^{v}=\left\{H\left(AT_{1}^{v}\right),H\left(AT_{2}^{v}\right),\ldots ,H\left(AT_{n}^{v}\right)\right\}$;
  3. Construct a polynomial $f\left(x\right)=H\left(AT_{1}^{v}\right)+H\left(AT_{2}^{v}\right)x+\ldots +H\left(AT_{n}^{v}\right)x^{n-1}\;\left(mod\;q\right)$, then pick *n* random points on the polynomial f(x) as a set: $P=\{\left(x_{i},y_{i}\right)|\left(x_{i},y_{i}\right)\in f\left(x\right)\wedge i=1\ldots n\}$;
  4. Construct two sequences $P_{x}$ and $P_{y}$ of all the $x_{i}$ and all the $y_{i}$ in *P*: $P_{x}=\left\{x_{i}|x_{i}\in f\left(x\right)\wedge \left(x_{i},f\left(x_{i}\right)\right)\in P\wedge i=1\ldots n\right\}$ and $P_{y}=\left\{y_{i}|y_{i}=f\left(x_{i}\right)\wedge x_{i}\in P_{x}\wedge i=1\ldots n\right\}$;
  5. Calculate the hash value of the sequence $P_{y}$: $PH_{y}=H\left(y_{1},y_{2},\ldots ,y_{n}\right),\;y_{1},y_{2},\ldots ,y_{n}\in P_{y}$;
  6. Send the challenge $P_{x}$ to $U_{A}$ through $CN_{pm}$. Note that $CN_{pm}$ should keep the $Ch=(PH_{y})$ to execute the following *Authorise* phase.
- **Response (pp,Ch):**
  $U_{A}$ generates the response Re to the challenge $P_{x}$ from $U_{B}$ via the following steps.
  1. Prepare the sequence of the identity attributes $AT^{\prime v}=\left\{AT_{1}^{\prime v},AT_{2}^{\prime v},\ldots ,AT_{n}^{\prime v}\right\}$ (shared by $U_{B}$) based upon $R_{n}\in Q$;
  2. Construct a new polynomial $g\left(x\right)=H\left(AT_{1}^{\prime v}\right)+H\left(AT_{2}^{\prime v}\right)x+\ldots +H\left(AT_{n}^{\prime v}\right)x^{n-1}\;\left(mod\;q\right)$;
  3. Take $P_{x}\in Ch$ to calculate the sequence $P_{y}^{\prime }=\left\{y_{i}^{\prime }|y_{i}^{\prime }=g\left(x_{i}\right)\wedge x_{i}\in P_{x}\wedge i=1\ldots n\right\}$ and then hash the sequence $P_{y}^{\prime }$: $PH_{y}^{\prime }=H\left(y_{1}^{\prime },y_{2}^{\prime },\ldots ,y_{n}^{\prime }\right),\;y_{1}^{\prime },y_{2}^{\prime },\ldots ,y_{n}^{\prime }\in P_{y}^{\prime }$;
  4. Send the response $Re=(PH_{y}^{\prime })$ to the consensus network $CN_{pm}$.
- **Authorise (Ch,Re):**
  The consensus network $CN_{pm}$ validates the two hash values in Ch and Re. If $PH_{y}\left(\in Ch\right)=PH_{y}^{\prime }\left(\in Re\right)$ holds (consensus check point), it means that the user $U_{A}$ can be authorised to access the requested data $B_{id}$ and the next phases are conducted; otherwise, the agent layer should deny the access request from $U_{A}$.
- **Encrypt ($pp,Q,AT^{v}$):**
  $U_{B}$ encrypts the requested data via the following steps.
  1. Acquire the requested data *M* based upon $B_{id}\in Q$ from $U_{A}$ and then calculate the hash value $H_{M}$ of the data *M*: $H_{M}=H\left(M\right)$;
  2. Generate a secure key $k\in Z_{q}$ for the symmetric encryption;
  3. Use AES to encrypt *M* with key *k* to get the ciphertext $C=AES_{k}\left(M,H_{M}\right)$. For decrypting $AES_{k}\left(M,H_{M}\right)$ to recover the plain data *M*, $AES_{k}^{\prime }$ is defined as the decryption process: $M=AES_{k}^{\prime }\left(C=AES_{k}\left(M,H_{M}\right)\right)$;
  4. Follow Section 3.2 to construct a polynomial $f^{*}\left(x\right)$ of degree *n* with *k* and $AT^{v}$: $f^{*}\left(x\right)=k+H\left(AT_{1}^{v}\right)x+H\left(AT_{2}^{v}\right)x^{2}+\ldots +H\left(AT_{n}^{v}\right)x^{n}\;\left(mod\;q\right)$;
  5. Generate a random integer $x_{p}\in Z_{q}$ and calculate a point $P(x_{p},y_{p}=f^{*}\left(x_{p}\right))$;
  6. Return (C,P) to $U_{A}$ through a secret channel.
- **Decrypt ($pp,C,P,AT^{\prime v}$):**
  $U_{A}$ can decrypt the ciphertext *C* after passing the *Authorise* phase via the following steps.
  1. Use the sequence $AT^{\prime v}$ organised in the former *Response* phase to construct a polynomial $g^{*}\left(x\right)$: $g^{*}\left(x\right)=a_{0}+H\left(AT_{1}^{\prime v}\right)x+H\left(AT_{2}^{\prime v}\right)x^{2}+\ldots +H\left(AT_{n}^{\prime v}\right)x^{n}\;\left(mod\;q\right)$. Note that $a_{0}$ is an unknown coefficient;
  2. Follow the Lagrange interpolation polynomial in the Section 3.2 to reconstruct the polynomial $g^{*}\left(x\right)$ fully, and then recover the key $k=g\left(0\right)=a_{0}\in Z_{q}$ for AES decryption with the point $P(x_{p},y_{p})$: $k=y_{p}-H\left(AT_{1}^{\prime v}\right)x_{p}-H\left(AT_{2}^{\prime v}\right)x_{p}^{2}-\ldots -H\left(AT_{n}^{\prime v}\right)x_{p}^{n}\;\left(mod\;q\right)$;
  3. Decrypt *C* to retrieve the plaintext $\left(M,H_{M}\right)=AES_{k}^{\prime }\left(C\right)=AES_{k}^{\prime }\left(AES_{k}\left(M,H_{M}\right)\right)$;
  4. If $H\left(M\right)=H_{M}$ holds, this algorithm outputs *M*; otherwise, it outputs ⊥.

## 6. Theoretical Analysis of CRA^3^

In this section, we first show the correctness of our proposed authorisation CRA^3^ scheme and then prove the confidentiality of CRA^3^. After that, the (data) integrity of CRA^3^ is illustrated in the third subsection, which is followed by a comparison of the security features in different blockchain-related authorisation schemes, as given in the last subsection.

### 6.1. Correctness

In the *Authorise* phase, if the data requester has the correct sequence of the identity attributes $AT^{\prime v}$, the condition $AT^{v}=AT^{\prime v}$ holds,

$AT^{v}=AT^{\prime v}$

$\Leftrightarrow \left\{H\left(AT_{1}^{v}\right),H\left(AT_{2}^{v}\right),\ldots ,H\left(AT_{n}^{v}\right)\right\}=\left\{H\left(AT_{1}^{\prime v}\right),H\left(AT_{2}^{\prime v}\right),\ldots ,H\left(AT_{n}^{\prime v}\right)\right\}$

⇔f(x)=g(x)

$\Leftrightarrow P_{y}=P_{y}^{\prime }\;\left(for\;the\;given\;P_{x}\right)$

$\Leftrightarrow PH_{y}=PH_{y}^{\prime }$.

This means that the data requester can pass the *Authorise* phase if and only if this requester has the correct corresponding sequence of the identity attributes for the requested blocks.

To satisfy the condition in the correctness definition, the authorised data requester should retrieve the key $k\in Z_{q}$ for AES decryption with the given point $P(x_{p},y_{p})$ on the polynomial f(x) in the *Decrypt* phase. Meanwhile, *P* should present on the correct reconstructed polynomial as well. Since the condition $\left\{H\left(AT_{1}^{v}\right),H\left(AT_{2}^{v}\right),\ldots ,H\left(AT_{n}^{v}\right)\right\}=\left\{H\left(AT_{1}^{\prime v}\right),H\left(AT_{2}^{\prime v}\right),\ldots ,H\left(AT_{n}^{\prime v}\right)\right\}$ holds after the *Authorise* phase, the reconstructed polynomial g(x) is the same as the original polynomial f(x) except for the unknown first coefficient $a_{0}=k$. Therefore, determining the secret key $g\left(0\right)=a_{0}=k\in Z_{q}$ for AES decryption requires only one point (shareholder) $P(x_{p},y_{p})$:

$k=a_{0}$

$=g\left(x_{p}\right)-AT_{1}^{\prime v}x_{p}-AT_{2}^{\prime v}x_{p}^{2}-\ldots -AT_{n}^{\prime v}x_{p}^{n}$

$=f\left(x_{p}\right)-AT_{1}^{\prime v}x_{p}-AT_{2}^{\prime v}x_{p}^{2}-\ldots -AT_{n}^{\prime v}x_{p}^{n}$

$=f\left(x_{p}\right)-AT_{1}^{v}x_{p}-AT_{2}^{v}x_{p}^{2}-\ldots -AT_{n}^{v}x_{p}^{n}$

=k(modq).

Hence, the authorised data requester can reconstruct the polynomial g(x) and restore the correct key *k* in the *Decrypt* phase to ensure $Decrypt(pp,C,AT^{\prime v})=M$ holds, where $C=Encrypt(pp,M,AT^{v})$.

### 6.2. Confidentiality (IND-CCA Security)

**Theorem** **1.** *According to Definition 1 above, the proposed CRA^3^ scheme is IND-CCA secure based on the Lagrange interpolating polynomial against the type-IND adversary in the random oracle model.*

*To be specific, let γ be a random oracle and $A_{1}$ be a Type-IND adversary with the advantage $Adv_{A_{1}}^{IND-CCA}$ against our proposed scheme. Hypothetically, $A_{1}$ requests a total of $Q_{\gamma }>0$ queries to the oracle γ; then there is an algorithm C that can determine all the correct coefficients for the given Lagrange interpolating polynomial with the advantage of at least $\frac{2}{Q_{\gamma }}Adv_{A_{1}}^{IND-CCA}$.*

**Proof.**  A polynomial f(x) with the sequence of the identity attributes $AT^{v_{i}}=\left\{AT_{1}^{v_{i}},AT_{2}^{v_{i}},\ldots ,AT_{n}^{v_{i}}\right\}\;\left(1⩽i⩽n\right)$ and a secure hash function $H:{\left\{0,1\right\}}^{*}\to {\left\{0,1\right\}}^{\lambda }$ consist of an instance of the Lagrange interpolating polynomial, where $f\left(x\right)=a_{0}^{i}+H\left(AT_{1}^{v_{i}}\right)x+H\left(AT_{2}^{v_{i}}\right)x^{2}+\ldots +H\left(AT_{n}^{v_{i}}\right)x^{n}$. The target data provider’s index is defined as *t*(1⩽t⩽n). The challenger C aims to determine $AT^{v_{t}}$ via executing $A_{1}$ as the subroutine. Next, C and $A_{1}$ play the game defined in Section 4.3.2.

- Initialise
  C first generates the public parameter pp=(q,H,AES) and then sends pp to $A_{1}$. After that, C generates *n* data providers (key-value pairs) $\left\{Rn^{i},AT^{v_{i}}|1⩽i⩽n\right\}$ and the target data provider is denoted by $\left\{Rn^{t},AT^{v_{t}}\right\}$. Note that all the generated $Rn^{i}\;\left(1⩽i⩽n\right)$ are given to the adversary $A_{1}$. Finally, C initialises one empty lists $List_{\gamma }$ and updates it continuously in the random oracle query *Identity attributes query*. If the same input is asked multiple times, the same answer will be returned.
- Queries
  C can respond to the queries requested by $A_{1}$ polynomial times in the following ways.
  1. *Identity attributes query (i)*: C generates the sequence of the identity attributes $rAT^{v_{i}}=\left\{rAT_{1}^{v_{i}},rAT_{1}^{v_{i}},\ldots ,rAT_{n}^{v_{i}}\right\}$ randomly and saves $\left(i,rAT^{v_{i}}\right)$ in $List_{\gamma }$ if it is the first time that *i* is queried. Then C respond with the sequence $rAT^{v_{i}}$. Otherwise, C should retrieve the sequence $rAT^{v_{i}}$ from $List_{\gamma }$ directly then return it to $A_{1}$.
  2. *Encrypt query(M,i)*: C uses the algorithm *Encrypt* to output the ciphertext $C=Encrypt(pp,M,AT^{v_{i}})$ and the point *P* (*P* should be on the polynomial constructed with $AT^{v_{i}}$ in the algorithm *Encrypt*).
  3. *Decrypt query(C,P,i)*: C tries to decrypt *C* via running $Decrypt(pp,C,P,AT^{v_{i}})$ then responds with the plain message. Note that there is a conditional branch caused by *i* to be discussed.
  If i=t, for each item $\left(i,rAT^{v_{i}}\right)$ in $List_{\gamma }$, C executes the operations.
  − Reconstruct the Lagrange interpolating polynomial $g^{*}\left(x\right)$ with $rAT^{v_{i}}$ and *P* to determine the secret key $k=a_{0}$ for AES decryption.
  − Recover $\left(M,H_{M}\right)$ by computing $AES_{k}^{\prime }\left(C\right)=AES_{k}^{\prime }\left(AES_{k}\left(M,H_{M}\right)\right)$.
  − If $H\left(M\right)=H_{M}$ holds, C returns *M* to $A_{1}$. If there is no item in the $List_{\gamma }$ that satisfies the condition, C returns ⊥ to $A_{1}$.
    If i≠t, C runs the $Decrypt(pp,C,P,AT^{v_{i}})$ algorithm directly and then sends the output to $A_{1}$ as the answer.
- Challenge
  $A_{1}$ submits two messages $M_{1}^{*},M_{2}^{*}\in {\left\{0,1\right\}}^{\lambda }$ with the same length to C, then C picks one random bit ρ from the set {0,1}. Finally, C computes the ciphertext $C^{*}$ of $M_{\rho }^{*}$ via the following steps:
  1. Choose a secret key $k\in Z_{q}$ for AES encryption and decryption;
  2. Determine $f^{*}\left(x\right)=k+H\left(AT_{1}^{v_{t}}\right)x+H\left(AT_{2}^{v_{t}}\right)x^{2}+\ldots +H\left(AT_{n}^{v_{t}}\right)x^{n}$;
  3. Pick a random point $P^{*}\left(x^{*},f^{*}\left(x^{*}\right)\right)$ on $f^{*}\left(x\right)$;
  4. Compute $C^{*}=AES_{k}\left(M_{\rho }^{*},H\left(M_{\rho }^{*}\right)\right)$.
  Finally, C sends the ciphertext $C^{*}$ and the point $P^{*}$ to the adversary $A_{1}$.
- *Constraints*
  1. $\left(M_{0}^{*},t\right)$ and $\left(M_{1}^{*},t\right)$ are not allowed to appear in the above *Encrypt query*;
  2. The target data provider’s index *t* and the challenge ciphertext $C^{*}$ are not allowed to appear in the above *Decrypt query*.
- Guess
  $A_{1}$ outputs one bit $\rho^{\prime }$ from the set {0,1}. At the same time, C picks a random element $\left(i,rAT^{v_{i}}\right)$ from $List_{\gamma }$ as the answer to the above given instance of the Lagrange interpolating polynomial.
- Probability analysis
  An event E is defined as that the adversary $A_{1}$ requests a query for the target sequence $AT^{v_{t}}$ in the *Identity attributes query* during the described game above. If the event E has happened, $AT^{v_{t}}$ occurs in at least one item of $List_{\gamma }$ at the end of this game.

However, if E does not happen, it means that $Pr\left[\rho^{*}=\rho^{{*}^{\prime }}|\neg E\right]=\frac{1}{2}$ holds. Meanwhile, the condition $Adv_{A_{1}}^{IND-CCA}⩽\left|Pr\left[\rho =\rho^{\prime }\right]-\frac{1}{2}\right|$ holds because of the definition of the type-IND adversary ($A_{1}$). Based upon the above analysis, the next two derivations can be illustrated.

$$\begin{array}{cc} Pr[\varphi =\varphi^{\prime }] & =Pr\left[\varphi =\varphi^{\prime }|E\right]Pr\left[E\right]+Pr\left[\varphi =\varphi^{\prime }|\neg E\right]Pr\left[\neg E\right]\\ \; & ⩽Pr\left[E\right]+Pr\left[\varphi =\varphi^{\prime }|\neg E\right]Pr\left[\neg E\right]\\ \; & =Pr\left[E\right]+\frac{1}{2}Pr\left[\neg E\right]\\ \; & =Pr\left[E\right]+\frac{1}{2}\left(1-Pr\left[E\right]\right)\\ \; & =\frac{1}{2}+\frac{1}{2}Pr\left[E\right]\\ Pr[\varphi =\varphi^{\prime }] & ⩾Pr\left[\varphi =\varphi^{\prime }|\neg E\right]Pr\left[\neg E\right]\\ \; & =\frac{1}{2}Pr\left[\neg E\right]\\ \; & =\frac{1}{2}-\frac{1}{2}Pr\left[E\right] \end{array}$$

Hence, we can deduce that the following derivation holds:

$$Adv_{A_{1}}^{IND-CCA}⩽\left|Pr\left[\rho =\rho^{\prime }\right]-\frac{1}{2}\right|⩽\frac{1}{2}Pr\left[E\right].$$

We can simplify this derivation such that $Pr\left[E\right]⩾2Adv_{A_{1}}.$

In conclusion, at the end of the game between the challenger C and the adversary $A_{1}$, the probability of the target sequence $AT^{v_{t}}$ being in the element(s) of $List_{\gamma }$ is at least $2Adv_{A_{1}}^{IND-CCA}$. Hence, the probability of generating the correct answer $\rho =\rho^{\prime }$ is at least $\frac{2}{Q_{\gamma }}Adv_{A_{1}}^{IND-CCA}$, where $Q_{\gamma }$ represents the total number of the elements in the list $List_{\gamma }$. □

### 6.3. Data Integrity

In our CRA^3^ scheme, the hash value $H_{M}=H\left(M\right)$ generated in the *Encrypt* algorithm can provide the data integrity of *M*. In the *Decrypt* algorithm, if the received *C* or *P* is incorrect or manipulated by the attacker in the communication between $U_{A}$ and $U_{B}$, the wrong *C* (or *P*) leads to the abnormal result of AES decryption result so that $\left(M,H_{M}\right)=AES_{k}^{\prime }\left(C\right)$ are incorrect (where $C=AES_{k}\left(M,H_{M}\right)$ and *k* is computed from *P*. Therefore, the condition $H\left(M\right)=H_{M}$ (step 4) cannot hold, which means our data integrity check can detect an abnormal *C* or *P* to protect the data integrity of *M*.

### 6.4. Comparison of Security Features

In Table 1, we compare the implemented security features of different blockchain-related authorisation schemes from the state-of-the-art of related work with that of our CRA^3^ scheme. It is clear that most of the compared schemes can support permissioned blockchains but CRA^3^ is the only one that can support an untrusted consensus network. Meanwhile, our CRA^3^ can also provide authorisation, confidentiality, and integrity for data access. However, in other compared schemes, the integrity feature is only implemented by [15] and no scheme considers confidentiality. The *Decentralizing Privacy* (DP) [12] scheme requires a database as a storage media; however, the DP scheme itself cannot support confidentiality or integrity.

## 7. Performance Evaluation and Results

The performance simulation and results are illustrated and discussed in this section. Two Raspberry Pi 2s [27] with Wi-Fi (as the mobile devices of the users $U_{A}$ and $U_{B}$) and one conventional computer with an Intel i5 processor running at 3.30 GHz (as a node of the consensus network in the permissioned blockchain) were used to perform the simulation. The local computational time efficiency for executing CRA^3^ was evaluated with respect to the time cost for transmitting encrypted data over Wi-Fi and the transaction fee (gas) of the consensus node in the simulation. Since there is yet no clear best practice to be used as a baseline for comparison, we selected an authorisation scheme for blockchain-based storage named *Decentralizing Privacy* (DP) [12] as our baseline. The authorisation supported by a trusted third party (TTP) in DP is policy-based but not anonymous, since the TTP knows the users’ identities. However, the designed authorisation in CRA^3^ is attribute-based and anonymous. Note that all the implemented experiments used the equivalent cryptographic security level (128-bit) [28], and that the transaction fee (gas) was calculated based upon the bytecodes generated by Ethereum Virtual Machine (EVM) [29] with PoA (Proof of Authority) [30] as the consensus mechanism.

**Figure 3:**
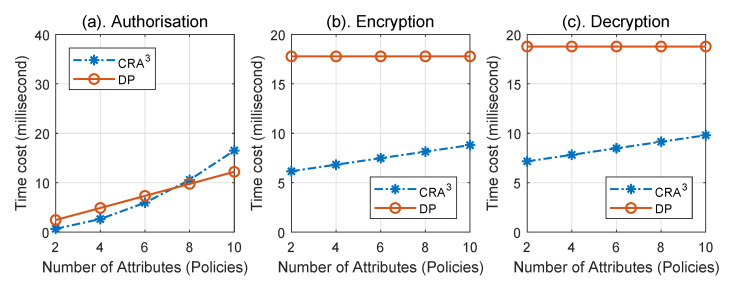
The time cost comparison of local computation on a Raspberry Pi 2 between CRA^3^ and DP (*Decentralise Privacy* scheme [12]).

First, the number of the attributes used for authorisation was varied from two to 10 in CRA^3^ (respective of policies in DP) to compare the time taken for local computation including authorisation, encryption, and decryption algorithms in the two schemes implemented on a conventional computer. The averaged results over 10 runs are shown in Figure 3. In the authorisation phase (Figure 3a), the time cost in both schemes increased with a similar trend when the number of attributes used was small. If the number of attributes used rose up to 10, our CRA^3^ scheme needed 25% more time to authorise the access when compared with the DP scheme. For the encryption and decryption phases, the time cost for the DP scheme kept stable whilst the time cost of the CRA^3^ scheme increased slowly, increasing with the number of attributes. On average, the time cost of the CRA^3^ scheme was 55% lower than that of the DP scheme; see Figure 3b,c.

**Figure 4:**
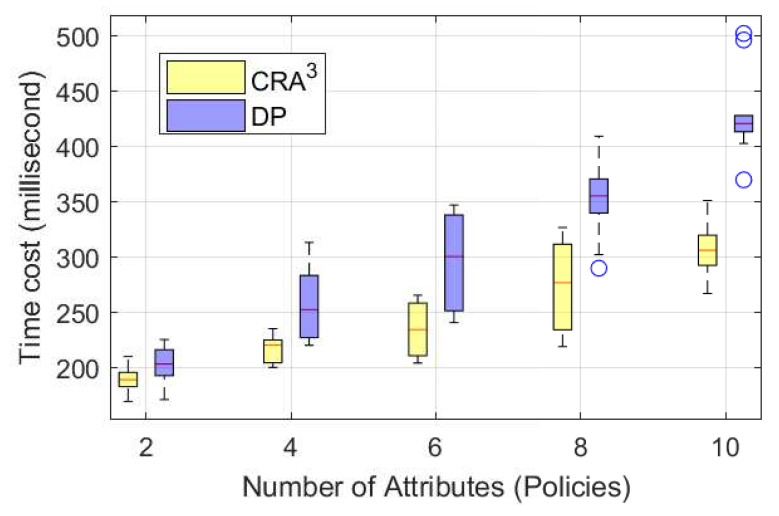
Comparison of the time costs of CRA^3^ and DP [12] for transmitting data over Wi-Fi.

Meanwhile, we measured the time cost for transporting data between users and $CN_{pm}$ (see Section 4.1) over Wi-Fi (Figure 4). The data included the attributes (i.e., policies) used for authorisation, the encrypted data (128 bytes) and the keys used for decryption in the two schemes. Since CRA^3^ only transmits two points in the *Authorise* phase whereas the DP scheme requires two policy lists for authorisation, the time consumption for transmitting data via Wi-Fi in CRA^3^ was about 24% lower than that in the DP scheme. Furthermore, the time cost in CRA^3^ had a lower growth rate when compared with the DP scheme.

**Figure 5:**
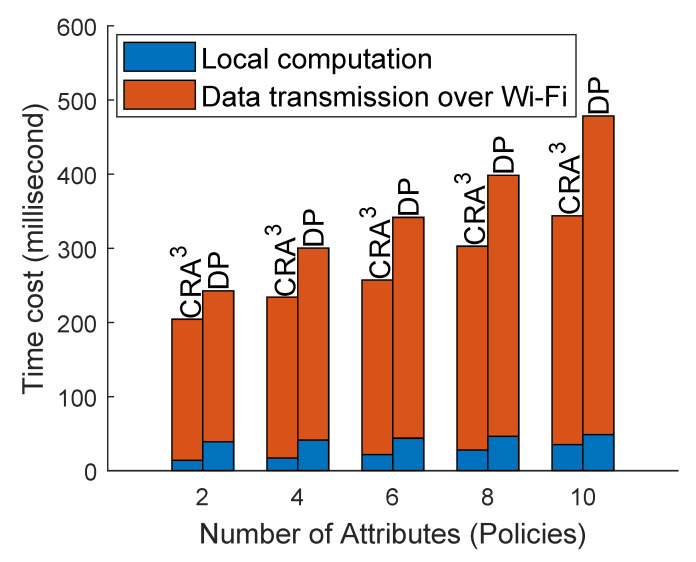
Comparison of total time cost of CRA^3^ and DP [12].

Thus, we summarise the total time cost of both local computation and data transmission via Wi-Fi in Figure 5. The total time cost in CRA^3^ was around 30% lower than that in the DP scheme. While the number of used attributes (i.e., policies) increased, the DP scheme consumed far more time than CRA^3^, in total.

**Figure 6:**
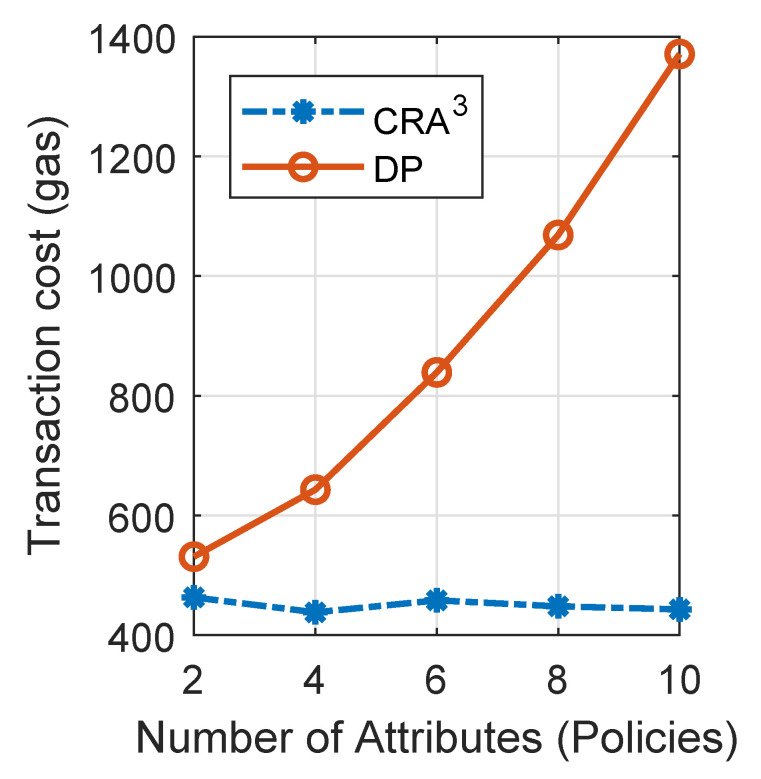
Transaction fee of CRA^3^ and DP [12] for authorisation.

Finally, the transaction fee (gas) for the *Authorise* phase performed in the consensus network was evaluated in a conventional computer (Figure 6). While the transaction fee of CRA^3^ kept stable (and was non-sensitive to the variation of used attributes), the transaction fee increased by the number of used policies in the DP scheme. This is because the DP scheme compares two policy lists in the transaction for authorisation but CRA^3^ only compares two points regardless of the number of used attributes.

## 8. Conclusions

In this paper, we proposed a privacy-enhanced authorisation CRA^3^ scheme under a consideration of untrusted nodes occurring in a consensus network of permissioned blockchain. Unlike existing work [10,12,16], CRA^3^ does not expose users’ credentials to the untrusted nodes in the consensus network for authorising data access. By applying CRA^3^ in a permissioned blockchain, users (data providers) can share private data with valid data requesters without leaking their private information. Therefore, CRA^3^ can help people to safeguard their privacy and prevent potential privacy leakage (e.g., caused by attackers) in permissioned blockchains. In terms of the communication overhead, CRA^3^ reduces the time cost for the communication during the authorisation since the size of the required data for authorising data access request is much smaller when compared with other methods. Furthermore, our consensus verification only relies on one equation and other computational work is executed by the data requester and receiver; hence, the consensus cost (transaction fee) is visibly cut down to save the user’s cost and the computational resource of the consensus network (i.e., lower workload) simultaneously.

## Figures and Tables

**Table 1:** Comparison of the security features in different blockchain-related authorisation schemes.

Scheme	Blockchain Type	Consensus Network Type	Security Features		
			Authorisation	Confidentiality	Integrity
[12]	Public/Permissioned	Trusted	√	× ^1^	× ^1^
[16]	Public	Trusted	√	×	×
[19]	Permissioned	Trusted	×	×	×
[15]	Permissioned	Trusted	√	×	√
CRA^3^ *	Permissioned	Trusted/Untrusted	√	√	√

^1^ The scheme depends on the deployed database to support the mentioned security feature. * CRA^3^: our proposed scheme, Challenge-Response Assisted Access Authorisation.
